# Isolated Left Ventricular Apical Hypoplasia, from Transient Neonatal Dysfunction to Maternal Hemodynamic Stress: A Comprehensive Review with Illustrative Cases

**DOI:** 10.3390/children13080983

**Published:** 2026-07-24

**Authors:** Mattia Pasquinucci, Martina Avesani, Anna La Rosa, Maria Elena Derchi, Davide Meneghesso, Davide Buffi, Federico Prefumo, Laura Tralli, Michela Marchesini, Anna Nocerino, Alessandra Grison, Claudia Santagati, Giulia Bordin, Gabriele De Tonetti, Elena Sofia Milandri, Giovanni Di Salvo, Gianluca Trocchio

**Affiliations:** 1 Department of Neuroscience, Rehabilitation, Ophthalmology, Genetics, Maternal and Child Health (DINOGMI), IRCCS Istituto Giannina Gaslini, 16147 Genoa, Italy; 2 Department of Pediatrics, AULSS 7 Pedemontana—San Bassiano Hospital, 36061 Bassano del Grappa, Italy; 3 Department of Paediatric Cardiology, Department of Woman’s and Child’s Health, Padua University Hospital, 35128 Padua, Italy; 4 Department of Women’s and Children’s Health, Padua University Hospital, 35128 Padua, Italy; 5 Cardiology Unit, Department of Surgical Sciences, IRCCS Istituto Giannina Gaslini, 16147 Genoa, Italy; 6 Obstetrics and Gynecology Unit, Department of Critical Care and Perinatal Medicine, IRCCS Istituto Giannina Gaslini, 16147 Genoa, Italy; 7 Department of Obstetrics, Gynecology and Fetal Medicine, AULSS 8 Berica—San Bortolo Hospital, 36100 Vicenza, Italy; 8 Department of Pediatrics and Neonatal Intensive Care, AULSS 8 Berica—San Bortolo Hospital, 36100 Vicenza, Italy; 9 Obstetric Anesthesia, Department of Critical Care and Perinatal Medicine, IRCCS Istituto Giannina Gaslini, 16147 Genoa, Italy; 10 Unit of Obstetrics and Gynecology, Santa Maria Delle Croci Hospital, AUSL Romagna, 48121 Ravenna, Italy

**Keywords:** isolated left ventricular apical hypoplasia, truncated left ventricle, ILVAH, spherical left ventricle, left ventricle hypoplasia, restrictive cardiomyopathy, banana-shaped right ventricle, ventricular septal defects, heart failure

## Abstract

**Highlights:**

**What are the main findings?**

- Isolated Left Ventricular Apical Hypoplasia (ILVAH) is frequently associated with long-term morbidity, with a significant proportion of reported cases (up to 69.5%) developing overt clinical symptoms such as heart failure or arrhythmias.
- The morphologically deficient and rigid left ventricle demonstrates distinct vulnerability to significant physiological shifts, specifically early postnatal afterload increases and rapid postpartum volume expansion.

**What are the implications of the main findings?**

- We hypothesize that ILVAH could be better conceptualized as a stress-sensitive restrictive congenital left ventricular disease that warrants proactive, life-long clinical surveillance, rather than a benign anatomical variant.
- Patients with ILVAH contemplating pregnancy require pre-conception counseling and structured, multidisciplinary planning with longitudinal cardiological follow-up, including serial echocardiographic monitoring, to anticipate and mitigate potential pregnancy-related events and complications, as well as postpartum acute decompensation.

**Abstract:**

**Background/Objectives**: Isolated Left Ventricular Apical Hypoplasia (ILVAH) is a rare congenital anomaly characterized by a truncated, spherical left ventricle (LV) with fibro-fatty apical replacement. Historically considered a benign condition of asymptomatic adults, its hemodynamic behavior under physiological stress remains poorly characterized. We present two distinct cases and a comprehensive literature review (n = 59) to redefine the clinical spectrum of ILVAH. **Methods**: To contextualize our findings, a comprehensive review of the literature was performed up to February 2026. We searched the PubMed/MEDLINE database using the terms “Isolated Left Ventricular Apical Hypoplasia”, “ILVAH”, “truncated left ventricle”, and “left ventricular apical hypoplasia”. The literature search and study selection were conducted in accordance with the PRISMA guidelines. **Case presentations**: Case 1 describes a male infant with ILVAH and muscular ventricular septal defects who unexpectedly developed transient systolic heart failure at one month of life, requiring prompt medical therapy (ACE inhibitors and diuretics) for functional recovery. Case 2 describes a 33-year-old woman with a known diagnosis of ILVAH. Serial echocardiography during her first pregnancy revealed progressive left atrial dilation and the onset of mild post-capillary pulmonary hypertension. Immediately following an elective cesarean section at 37 weeks, she experienced acute heart failure. She was successfully managed with pre-emptive volume offloading. **Conclusions**: ILVAH is not a universally benign anomaly. The morphologically deficient and rigid ventricle is vulnerable to afterload shifts in infancy and rapid volume expansion in adulthood. A review of all previously reported pregnancies in ILVAH reveals a consistent pattern of severe, unrecognized postpartum pulmonary edema. Proactive, multidisciplinary management is suggested to prevent clinical decompensation. Our findings suggest that ILVAH may act as a stress-sensitive restrictive congenital left ventricle disease rather than a universally benign anatomical variant, a hypothesis that warrants further investigation.

## 1. Introduction

Isolated Left Ventricular Apical Hypoplasia (ILVAH) is a rare congenital heart disease, first described by Fernandez-Valls et al. in 2004 using cardiac magnetic resonance (CMR) imaging as the gold standard for diagnosis [1]. It is defined by four main morphological features: a truncated, spherical left ventricle (LV) with an absent or hypoplastic apex; abnormal papillary muscle architecture; apical fat replacement; and elongation of the right ventricle (RV) encircling the deficient LV apex.

Historically, ILVAH has been considered a relatively benign anomaly, often diagnosed incidentally in asymptomatic adults [2,3]. However, its true natural history and clinical spectrum remain largely undefined. Emerging evidence suggests that the morphologically altered LV may be vulnerable to specific hemodynamic stressors, leading to decompensation. Currently, only a few reports describe the behavior of ILVAH during significant physiological shifts, and only three cases of patients with ILVAH undergoing pregnancy have been documented [4,5,6].

In this paper, we aim to illustrate the dynamic clinical spectrum of ILVAH by reporting two distinct cases managed in two Italian centers: an infant presenting with transient neonatal heart failure (HF), and a 33-year-old woman experiencing acute postpartum decompensation. Furthermore, we provide a comprehensive literature review focusing on the hemodynamic challenges of pregnancy in ILVAH, comparing our patient’s clinical course with previously reported cases to propose tailored management strategies.

## 2. Materials and Methods

### 2.1. Search Strategy and Data Extraction

**Figure 1:**
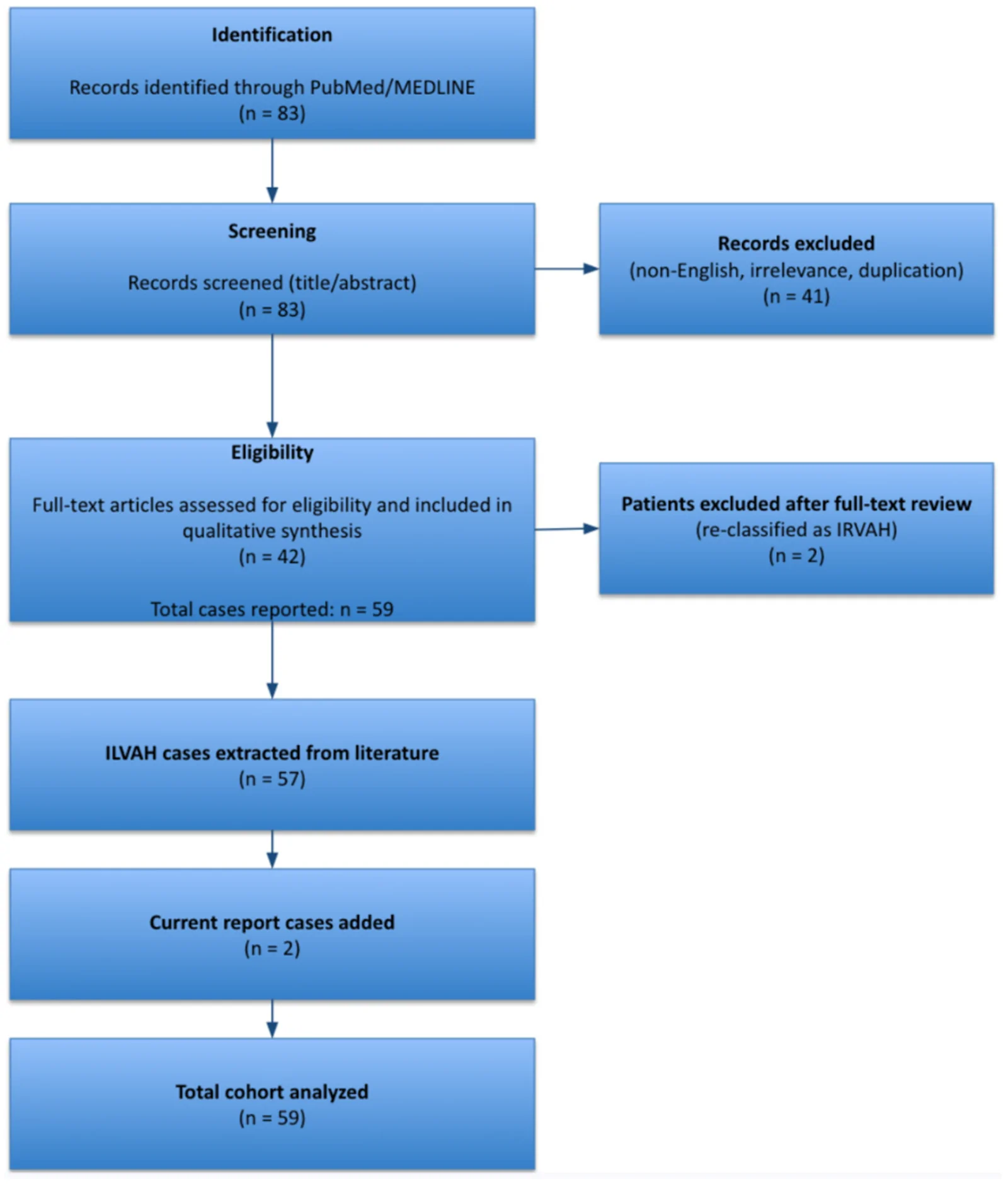
PRISMA flow diagram detailing the literature search strategy and study selection process.

A comprehensive review of the English-language literature was performed from January 2004 up to February 2026. We searched the PubMed/MEDLINE database using the terms “Isolated Left Ventricular Apical Hypoplasia”, “ILVAH”, “truncated left ventricle”, and “left ventricular apical hypoplasia”. The literature search and study selection were conducted in accordance with the PRISMA (Preferred Reporting Items for Systematic Reviews and Meta-Analyses) guidelines (Figure 1).

A dedicated database was constructed to extract demographic characteristics, age at diagnosis, clinical presentation, associated morphological anomalies, and therapeutic management of all reported cases. A sub-analysis was specifically performed to isolate and compare all female patients who experienced pregnancy, focusing on the timing of diagnosis (pre-conception vs. post-partum), clinical symptoms, and maternal outcomes.

### 2.2. Ethics Statement

The research reported in this paper adhered to the PRISMA guidelines. The study was conducted in accordance with the ethical standards laid down in the Declaration of Helsinki. The authors confirm that patient consent forms have been obtained for this article. Ethics Committee approval was waived by the local IRB due to the report of fully anonymized data.

### 2.3. Statistical Analysis

Continuous variables were expressed as mean and standard deviation or median and interquartile range, according to the data distribution. The data were analyzed using the Shapiro–Wilk test to verify normal distribution. Categorical variables were presented as absolute numbers and percentages.

## 3. Case Presentation

### 3.1. Case 1

**Figure 2:**
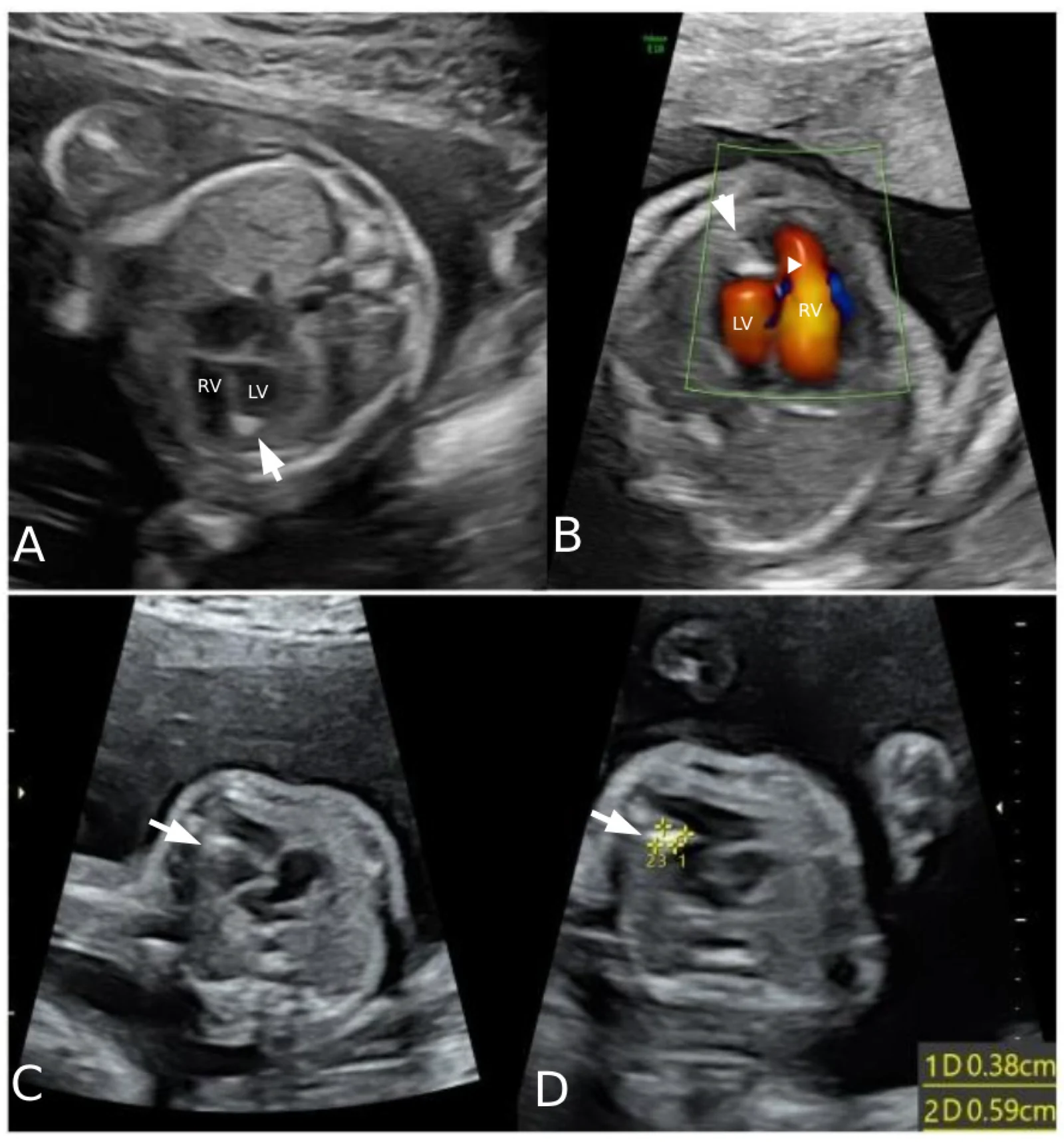
(**A**) Four-chamber view at 20 weeks of gestation revealing a distinct, highly echogenic triangular mass (white arrow) occupying the anatomical apex of the left ventricle (LV). (**B**) Color Doppler evaluation at 20 weeks: the LV intracavitary flow abruptly ceases proximal to the space-occupying lesion (white arrow), leaving the apex unfilled. Notably, the right ventricular (RV) apex exhibits normal flow and lacks the characteristic “banana-shaped” elongation at this early stage. A small apical ventricular septal defect (white triangle) can be seen. (**C**) Four-chamber view at 27 weeks of gestation showing the persistence of the apical lesion (white arrow) and the initial signs of ventricular remodeling. (**D**) Detailed measurement (yellow crosses) of the focal apical mass at 27 weeks (approximately 3.8 × 5.9 mm); the lesion appears increasingly integrated into the adjacent myocardium.

A male infant was born at term (37 weeks + 2 days) via cesarean section due to intrauterine growth restriction and breech presentation. A prenatal ultrasound at 20 weeks had raised suspicion of a cardiac anomaly, describing a “hyperechogenic formation” at the left ventricular apex. An apical muscular ventricular septal defect (VSD) was also suspected. The ultrasound findings were subsequently confirmed by fetal echocardiography, which revealed the presence of a distinctly hyperechogenic rounded formation of approximately 3.5 mm located in the left ventricle at the apical septal region. This formation did not protrude into the ventricular chamber and did not interfere with outflow or valvular function. During pregnancy, serial echocardiographic follow ups were performed at 20, 24 and 34 weeks (Figure 2). The ultrasound findings changed over the course of these evaluations, with the formation appearing less hyperechogenic and less well defined at the last examination, seemingly arising from the myocardium at the 34-week scan.

**Figure 3:**
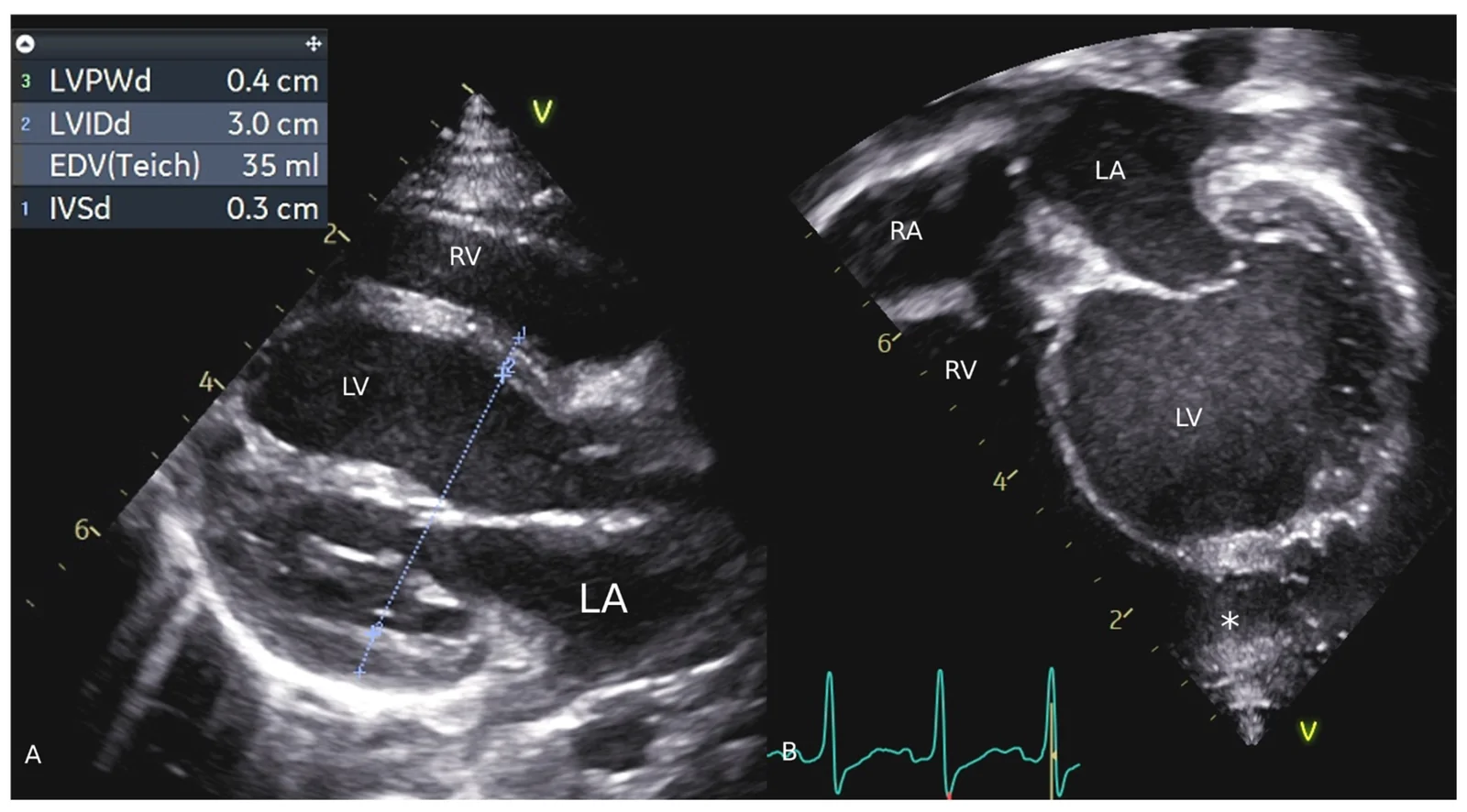
(**A**) Parasternal long-axis (PLAX) and (**B**) apical four-chamber views showed the hallmark morphological feature of ILVAH: a blunt and truncated, spherical geometry of the left ventricular apex, while the right ventricle showed a “banana-shaped” (denoted by *) elongated geometry wrapping around the distal left ventricle.

Postnatal echocardiography confirmed the presence of congenital cardiac anomalies. The LV appeared spherical with a truncated, hyperechogenic apex failing to reach the anatomical apex, despite a preserved left ventricular ejection fraction (LVEF). The RV was elongated, wrapping around the distal LV (Figure 3).

**Figure 4:**
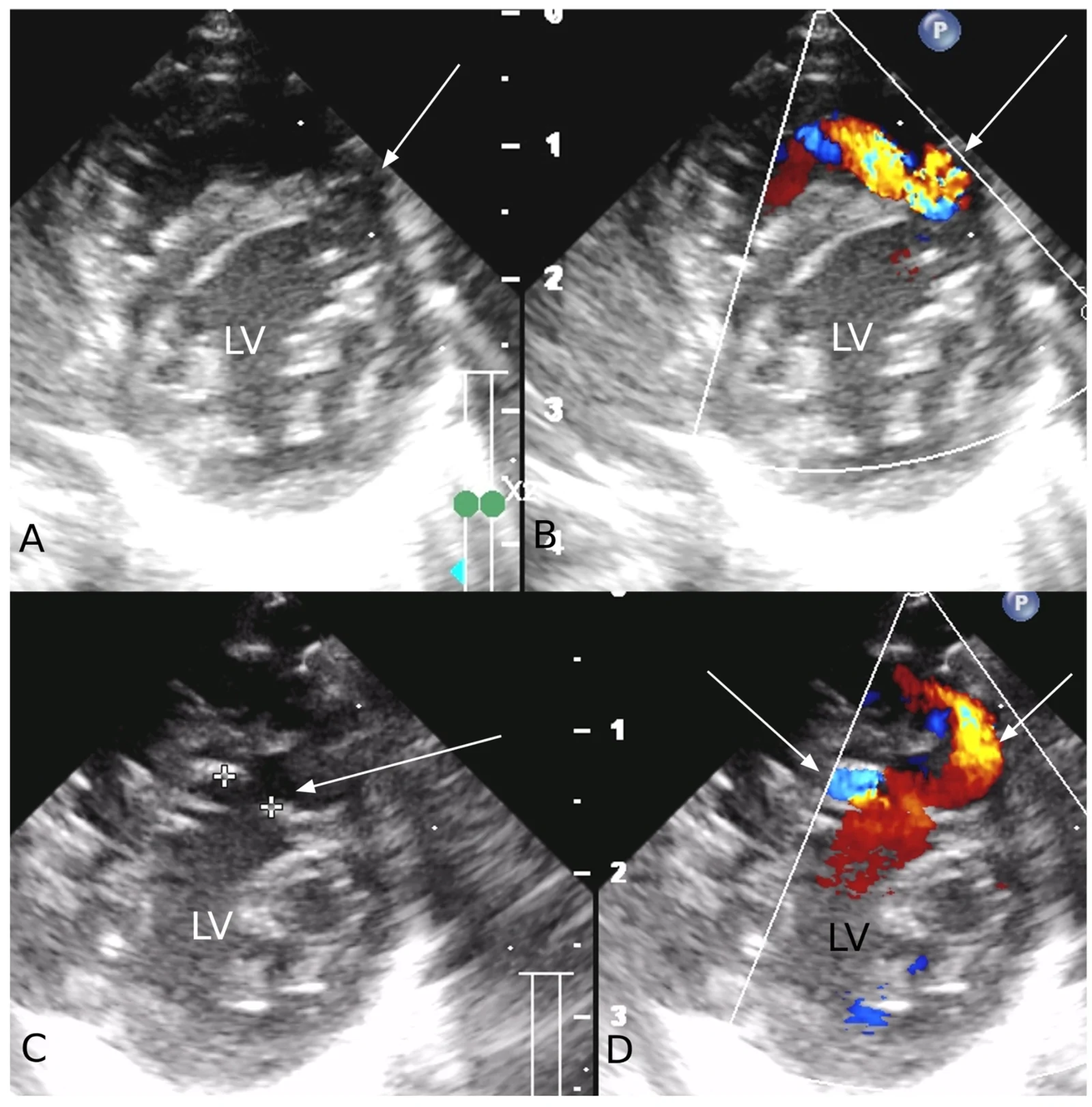
Parasternal short-axis (PSAX) echocardiography detailing multiple small muscular ventricular septal defects (VSDs) within the trabecular meshwork (white arrows). (**A**) B-mode evaluation showing the structural discontinuity in the interventricular septum. (**B**) Corresponding Color Doppler assessment demonstrating high-velocity systolic shunting jets across the defects. (**C**) Detailed B-mode magnification showing the exact localization and measurement of the defects using electronic calipers (white crosses, +). (**D**) Additional Color Doppler confirmation of the multiple distinct restrictive left-to-right shunts. LV = Left Ventricle.

Three small apical muscular VSDs were also identified (Figure 4).

**Figure 5:**
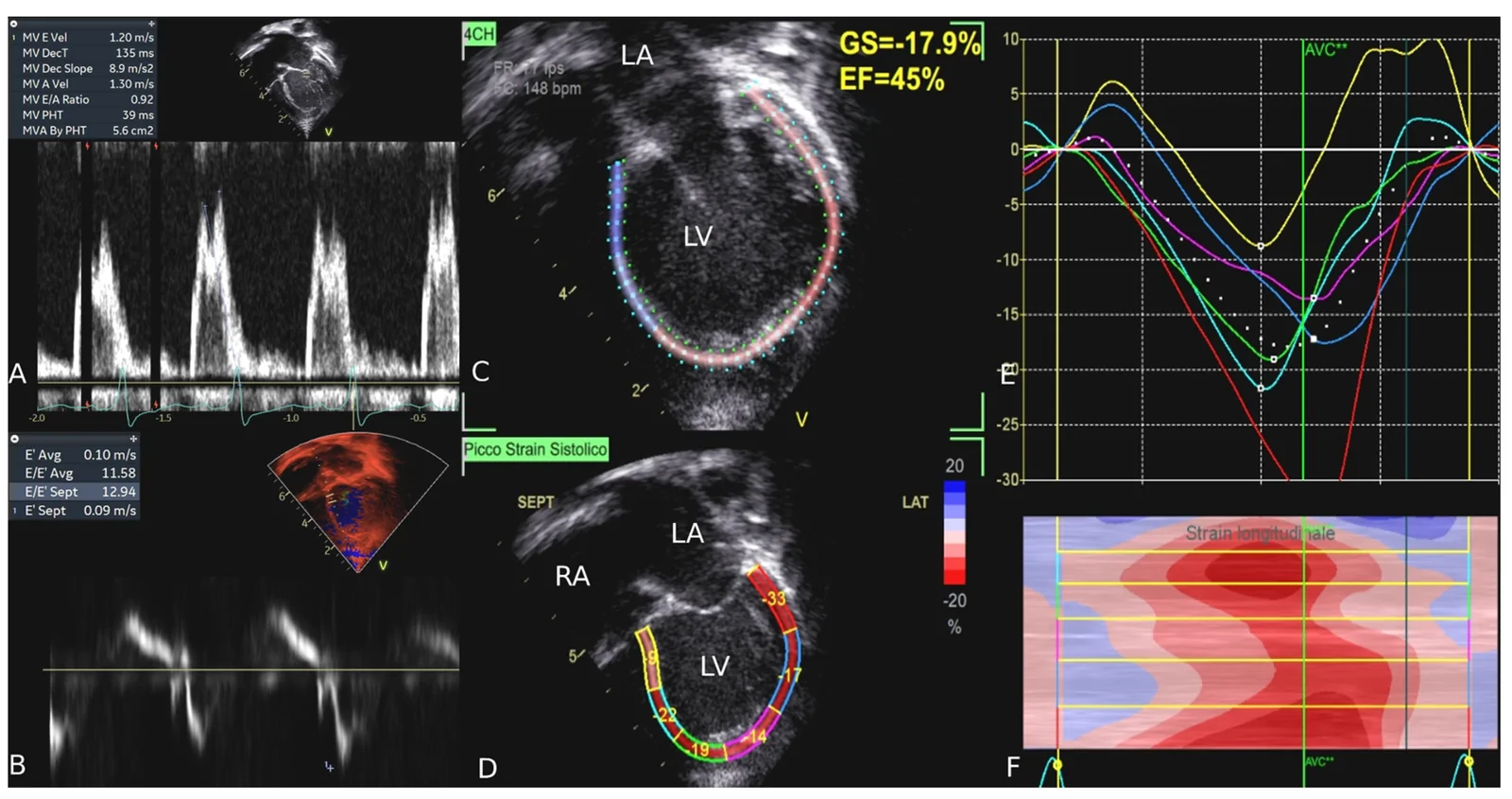
Comprehensive echocardiographic functional assessment during the acute heart failure presentation (Case 1). (**A**) Transmitral pulsed-wave Doppler showing an E/A ratio of 0.9. (**B**) Tissue Doppler imaging (TDI) demonstrating an E/E’ ratio of 12. (**C**) Apical four-chamber view with Speckle-Tracking Echocardiography (STE) overlay, highlighting the characteristic spherical, truncated left ventricular (LV) apex. (**D**–**F**) Longitudinal strain curves demonstrating mildly reduced global longitudinal strain (GLS = −17.9%) and an ejection fraction of 45%. The strain analysis reveals a regional base-to-apex functional gradient. RV = Right Ventricle; LA = Left Atrium.

In the following weeks, the patient developed clinical signs of HF, with tachypnea, tachycardia and failure to thrive. Laboratory findings revealed elevated myocardial stress markers (NT-proBNP 2317 ng/L) and minimal troponin release (High-Sensitivity Troponin I 81 ng/L). Echocardiography documented a significant drop in systolic function, with a Left Ventricular Ejection Fraction (LVEF) of 45%, and left atrial volume at the upper limit of normal (LAVi 19 mL/m^2^). The E/A and E/E’ average were 0.9 and 12, respectively (Figure 5).

Medical therapy was promptly initiated with loop diuretics (Furosemide 0.7 mg/kg/day, administered in two divided doses) and ACE inhibitors (Captopril 0.3 mg/kg/day, administered in three divided doses; later transitioned to Enalapril 0.25 mg/kg/day, administered in two divided doses). Over the following months, the patient exhibited an excellent clinical response with complete recovery of systolic function. At the 1-year follow-up, echocardiography demonstrated normalization of LVEF (59%) despite a persistently globular left ventricular geometry. However, global longitudinal strain (GLS) from the four-chamber view and indices of diastolic function remained impaired (GLS −17.9%, E/A 0.9, E/E′ 12). Right ventricular function was preserved (TAPSE 23 mm).

**Figure 6:**
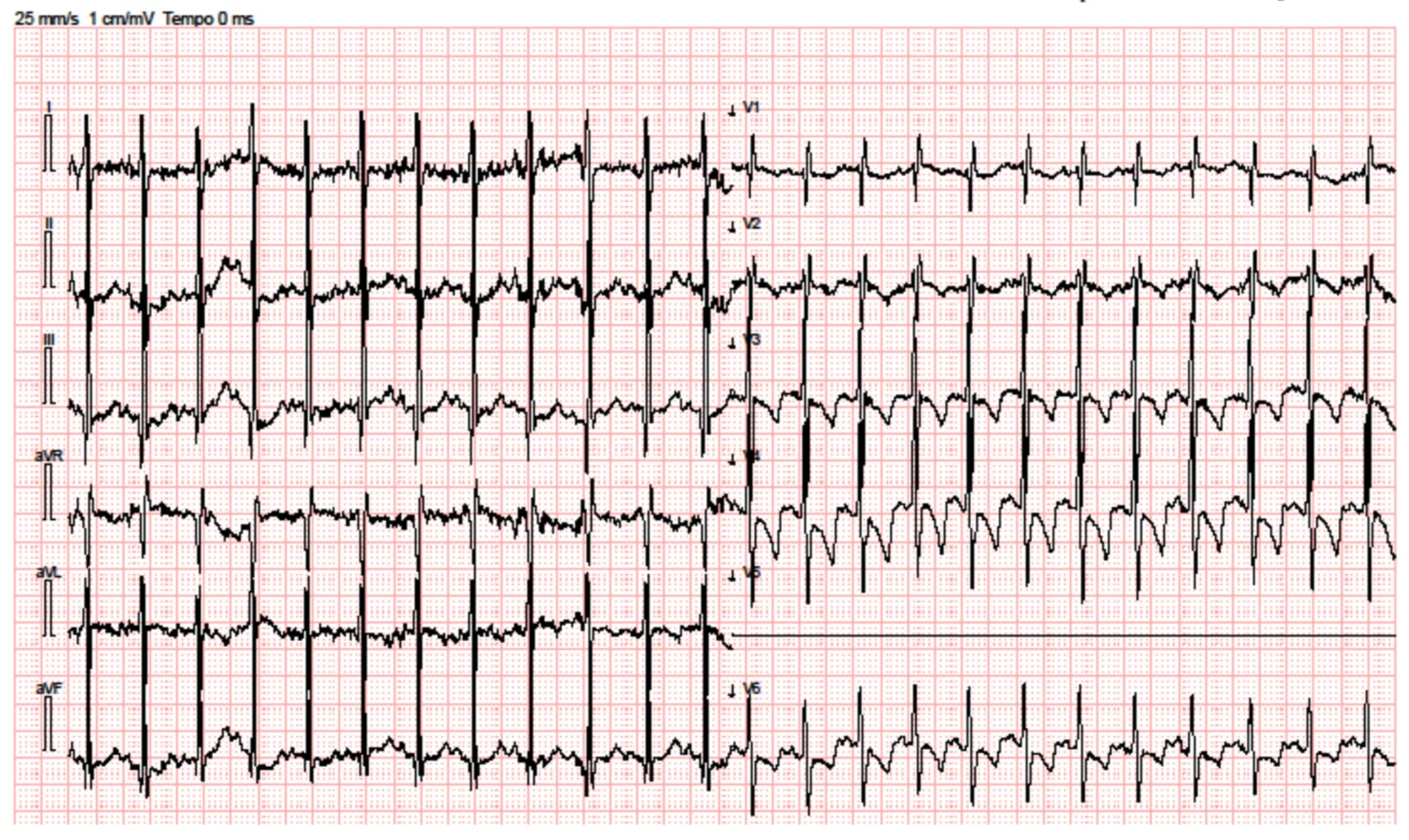
Twelve-lead electrocardiogram (ECG) showing sinus rhythm with negative T waves extending to lead V6.

The infant is currently asymptomatic with appropriate growth, although the electrocardiogram (ECG) continues to show negative T waves extending to lead V6 (Figure 6).

### 3.2. Case 2

**Figure 7:**
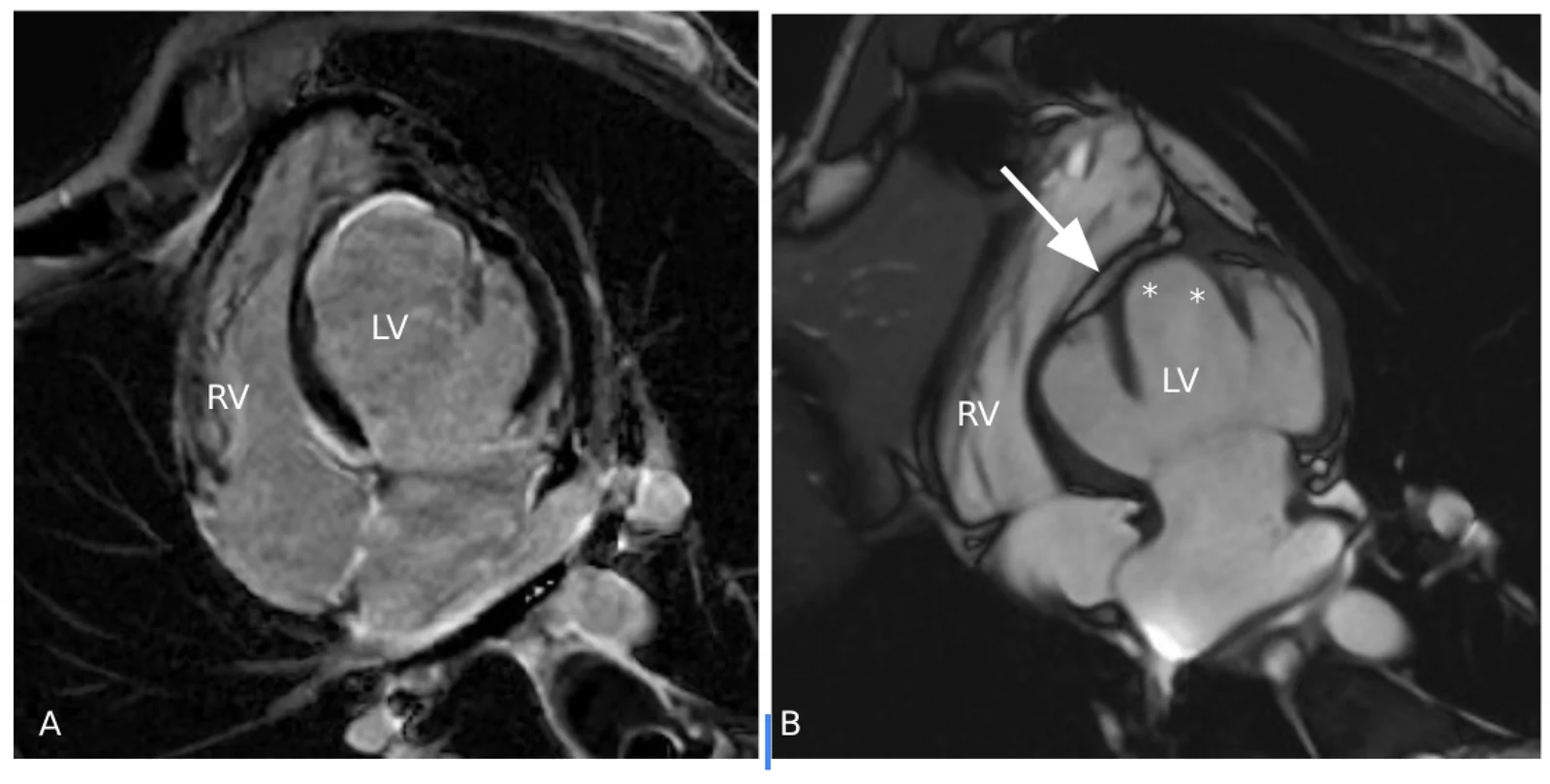
Cardiac magnetic resonance (CMR) four-chamber images illustrating the pathognomonic morphological hallmarks of ILVAH. (**A**) Phase-sensitive inversion recovery (PSIR) and (**B**) cine balanced steady-state free precession (bSSFP) images demonstrate a truncated, spherical left ventricle (LV) with failure of normal apical formation, apically displaced papillary muscles (denoted by *), and an elongated, banana-shaped right ventricle (RV) wrapping around the deficient LV. On cine bSSFP imaging, the LV apical fatty tissue exhibits intrinsically high signal intensity (white arrow), corresponding to the region of extensive late gadolinium enhancement (LGE) on the corresponding PSIR image.

A 33-year-old woman (gravida 1, para 0) with ILVAH was referred to our center for multidisciplinary pregnancy management. Her condition was originally diagnosed during adolescence (age 17) via CMR, initially performed for suspected dilated cardiomyopathy. The CMR revealed a normal-volume, spherical LV (EDV 81 mL/m^2^) with a truncated apex showing apical akinesia (Figure 7). Additional tissue characterization demonstrated transmural late gadolinium enhancement (LGE) and T1 alterations consistent with fibro-fatty replacement.

**Figure 8:**
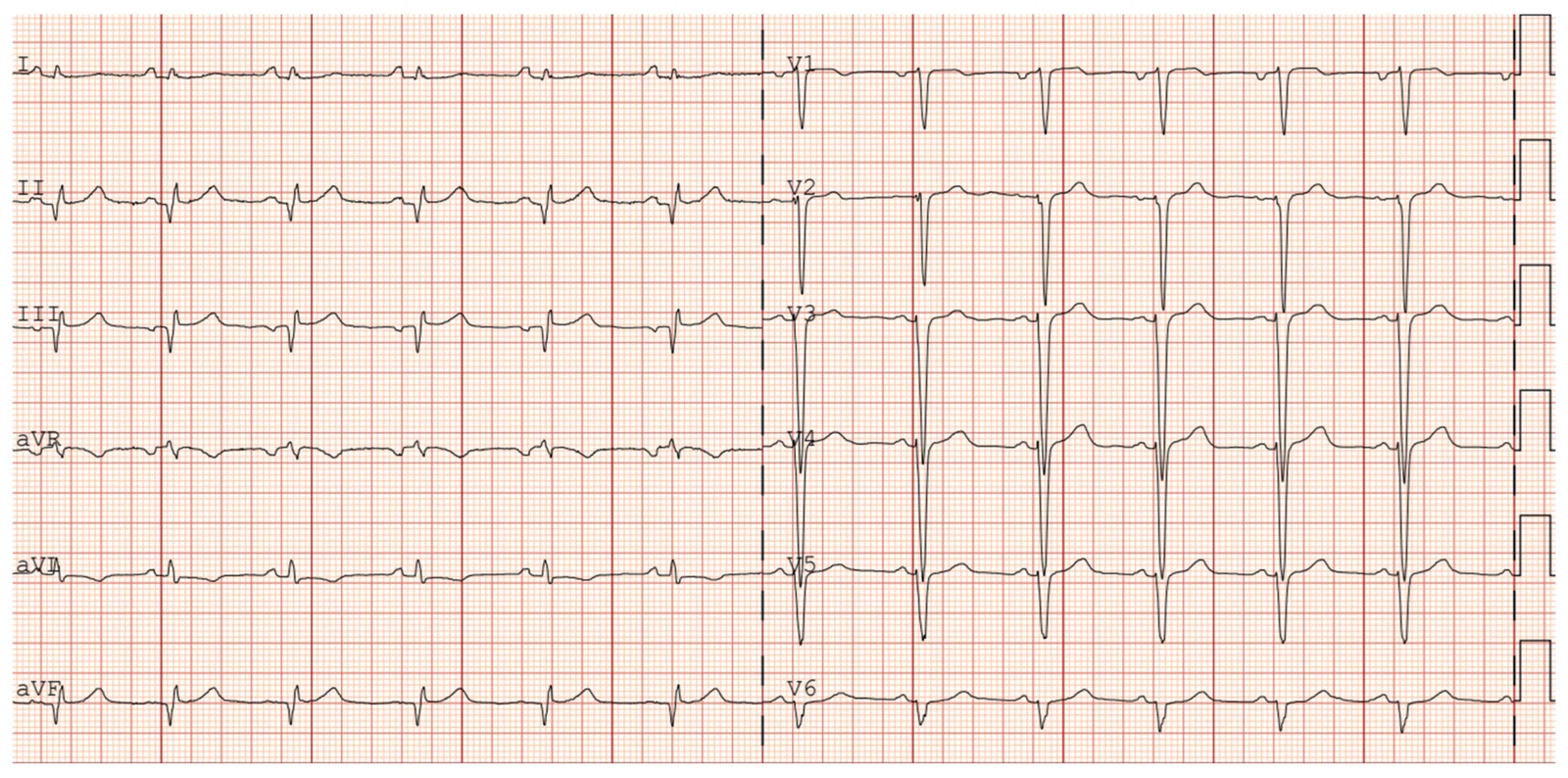
Twelve-lead electrocardiogram (ECG) showing sinus rhythm with poor R-wave progression, minor intraventricular conduction delay, and Q waves in the inferior leads (II, III, and aVF).

The ECG showed poor R-wave progression in the precordial leads, minor intraventricular conduction delay, and Q waves in the inferior leads (Figure 8).

While the patient remained in NYHA class I–II, serial echocardiographic monitoring during gestation revealed progressive hemodynamic changes throughout pregnancy. The left atrial (LA) area steadily increased from a pre-pregnancy baseline of 20 cm^2^ to 22 cm^2^ (parasternal long-axis diameter 44 mm) at 30 weeks of gestation, and further to 26 cm^2^ at 34 weeks. Concurrently at 34 weeks, she developed signs of mild, newly onset pulmonary hypertension (PH), likely post-capillary in nature, reflecting retrograde pressure transmission from the non-compliant LV. By 37 weeks (prior to delivery), the LA area had peaked at 33 cm^2^ (parasternal long-axis diameter 51 mm).

**Figure 9:**
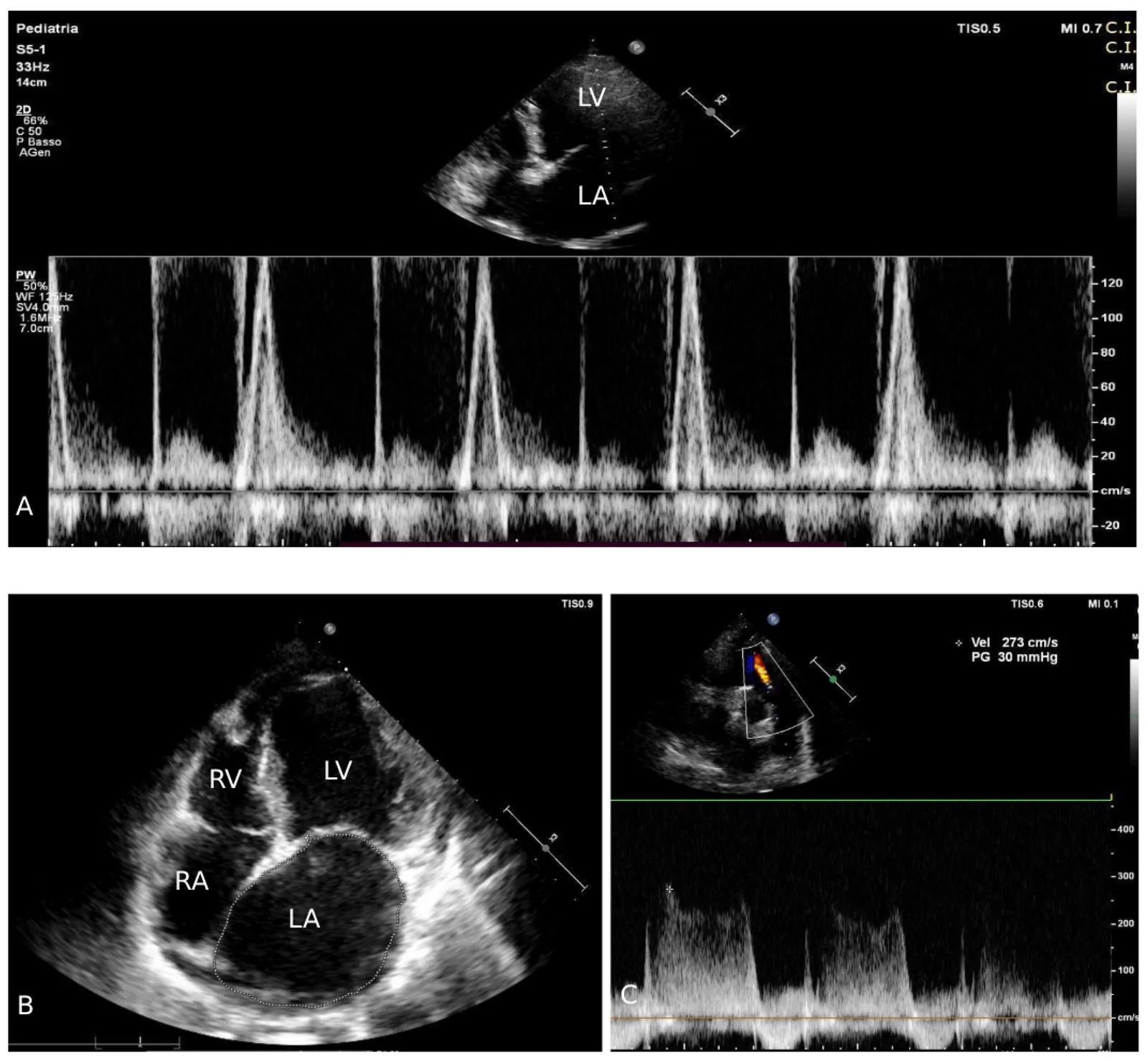
Transthoracic echocardiography (TTE) performed during the acute postpartum decompensation (Case 2). (**A**) Transmitral pulsed-wave Doppler demonstrating a restrictive filling pattern, with an E/A ratio of 2.4. (**B**) Apical four-chamber view highlighting significant left atrial enlargement, reflecting the retrograde transmission of elevated filling pressures from the rigid, truncated left ventricle. (**C**) Continuous-wave Doppler of the pulmonary regurgitation envelope used to estimate the mean pulmonary artery pressure (mPAP), confirming mild post-capillary pulmonary hypertension.

She underwent an elective cesarean section at 37 weeks. As anticipated by the third-trimester echocardiographic trend, the immediate postpartum period was complicated by acute HF. NT-proBNP levels rapidly escalated from 512 ng/L (Day 1 post-partum) to 1279 ng/L (Day 3), accompanied by clinical signs of decompensation. Global systolic function remained preserved (LVEF 50–55%). Cardiac ultrasound findings can be found in Figure 9.

Management focused on strict fluid restriction (maximum 1 L/day) and offloading the ventricle. She was started on Furosemide (up to 12.5 mg TID) and low-dose Enalapril (titrated to 2.5 mg/day). This resulted in rapid diuresis, clinical stabilization, and a significant drop in natriuretic peptides. She was discharged on postpartum day 11 with a normalized fluid status. At the 5-month follow-up, she remains stable and asymptomatic on Enalapril 2.5 mg once per day, with echocardiography showing reduced left atrial dimensions, resolution of PH and stable LV systolic parameters.

## 4. Discussion

ILVAH is an extremely rare congenital anomaly. To date, our comprehensive review identified 57 reported cases of ILVAH [7,8,9,10,11,12,13,14,15,16,17,18,19,20,21,22,23,24,25,26,27,28,29,30,31,32,33,34,35,36,37,38,39,40,41,42]. We grouped these patients with the 2 cases described above; the data can be found in Table 1.

Analysis of this cohort challenges the traditional perception of ILVAH as a universally benign, isolated structural curiosity. The median age at diagnosis is 26 years, with an equal gender distribution (female sex 45%). Notably, while early literature emphasized asymptomatic presentations, our data reveal that a significant proportion of patients (41/59, 69.5%) ultimately develop overt clinical symptoms, primarily driven by heart failure or arrhythmogenic events. The most frequently reported symptoms were dyspnea (n = 24), chest discomfort (n = 15), fatigue (n = 10), palpitations (n = 7), dizziness/syncope (n = 3), and cardiac arrest (n = 2). At the time of diagnosis, baseline LVEF was impaired in 31 cases (out of 43 with available data), mean 46%, necessitating standard heart failure pharmacotherapy in 26 patients of the overall cohort (data available for n = 50). Furthermore, the nomenclature of “isolated” hypoplasia is frequently a misnomer; associated morphological anomalies, such as interatrial or interventricular shunts and atrioventricular valve dysplasia, were documented in 11 patients. Crucially, electrical abnormalities are a hallmark of the condition. Electrocardiographic abnormalities were nearly ubiquitous, being present in 87.5% of cases, ranging from non-specific T-wave inversions to conduction delays (Table 2).

Beyond structural ECG changes, clinically significant arrhythmias—including atrial fibrillation, sustained ventricular tachycardia, and sudden cardiac arrest—were reported. These findings establish ILVAH as a complex congenital heart disease with substantial long-term morbidity, heavily dependent on the fibro-fatty arrhythmogenic substrate and the progressive loss of ventricular compliance. The two cases presented in this report highlight the broad and unpredictable clinical spectrum of ILVAH across different stages of life, challenging the historical assumption that the condition is universally benign.

### 4.1. Early Presentation and Transient Dysfunction

While adult diagnoses predominate, our Case 1 demonstrates that ILVAH can manifest clinically during infancy. The presence of multiple VSDs within the trabecular meshwork is a recognized, albeit rare, associated defect of the disease. However, the transient decline in LVEF at one month of life is a novel finding. We hypothesize that this early dysfunction likely reflects a mismatch between the postnatal increase in afterload and reduced ventricular compliance.

The rapid response to ACE inhibitors and diuretics suggests that pediatric cases warrant close early monitoring rather than simple watchful waiting.

### 4.2. Pregnancy and Hemodynamic Stress

The most critical insights emerge from our Case 2, which represents, to the best of our knowledge, the fourth reported case of pregnancy in a patient with ILVAH, but crucially, the first case with a known pre-conception diagnosis. A review of the three previously reported pregnancies, described by Flett et al. [4], Patrianakos et al. [5] and Ding et al. [6], reveals a stark contrast in clinical trajectories (Table 3).

All three historical cases were diagnosed retrospectively in the post-partum period following severe clinical presentations, predominantly peripartum pulmonary edema and post-capillary pulmonary hypertension. The severity of their decompensation necessitated aggressive interventions, including complex heart failure regimens, Amiodarone, Coumadin, and, in one instance, Implantable Cardioverter Defibrillator (ICD) placement.

Conversely, our patient was prospectively monitored. She remained asymptomatic throughout the gradual volume expansion of the second and third trimesters, although serial echocardiographic assessments documented progressive left atrial enlargement during pregnancy. Progressive left atrial enlargement may serve as an early warning marker of impending decompensation. However, despite the optimal status prior to delivery, she developed mild acute heart failure (NT-proBNP peak 1392 pg/mL) immediately post-partum. This consistent timing across all four known cases provides a crucial pathophysiological clue. The decompensation is likely driven by the sudden post-partum autotransfusion (fluid shift from the contracting uterus) into the systemic circulation. In ILVAH, the structural absence of the apex is replaced by non-compliant, fibro-fatty tissue. This creates a stiff, restrictive LV physiology that cannot accommodate rapid volume loading, leading to elevated filling pressures and left atrial dilation.

The comparison between our proactively managed case and the historical cohort emphasizes that women with ILVAH who desire pregnancy may benefit from multidisciplinary pregnancy planning and structured clinical and echocardiographic surveillance during gestation. Pre-conception counseling should be offered.

Our experience suggests that decompensation might not be entirely incidental. We hypothesize that it could be anticipated and that its severity could be mitigated with prompt recognition of post-partum volume shifts. In our patient, acute heart failure was managed successfully using prompt, low-dose diuretic and vasodilator therapy, avoiding the need for aggressive advanced heart failure therapies.

### 4.3. Diastolic Restriction and the Pathway to Pulmonary Hypertension

The serial echocardiographic monitoring of our pregnant patient provides an unprecedented, real-time window into the hemodynamic failure mechanism of ILVAH. During normal pregnancy, maternal plasma volume expands by up to 50%, peaking around 32–34 weeks of gestation. In a healthy heart, the left ventricle easily accommodates this preload through enhanced diastolic distensibility. Conversely, the extensive fibro-fatty replacement of the LV apex in ILVAH creates a rigid, non-compliant chamber.

Unable to accommodate the physiological volume load, LV diastolic filling pressures rise and are transmitted retrogradely. This was meticulously documented in our patient by the progressive, massive dilation of the left atrium (from 20 cm^2^ pre-conception to 33 cm^2^ at term) and the onset of de novo post-capillary pulmonary hypertension exactly at the 34-week peak of volume expansion.

This pathophysiological cascade perfectly explains the outcomes reported in the historical cohort. The patients described by Flett, Patrianakos, and Ding [4,5,6] all presented with peripartum pulmonary edema. By intercepting this trajectory through close monitoring and recognizing the early signs of retrograde congestion (LA dilation and PH), we likely anticipated the postpartum decompensation. This confirms that the primary physiological threat of ILVAH in pregnancy is diastolic heart failure, mandating serial evaluations long before the onset of overt clinical symptoms.

### 4.4. Arrhythmogenic Diathesis and the Fibro-Fatty Substrate

Beyond the mechanical constraints of a truncated ventricle, ILVAH carries a significant, intrinsic arrhythmogenic burden [4,6,8,13,15,17,21,27,31,33,35,36,37,39,41]. The morphological hallmark of this condition is apical fibro-fatty replacement. This fibrous tissue creates an ideal anatomical and electrophysiological substrate for re-entrant tachyarrhythmias, bearing pathophysiological similarities to arrhythmogenic cardiomyopathies.

A review of the literature reveals a notable prevalence of both atrial and life-threatening ventricular arrhythmias, occasionally culminating in sudden cardiac arrest (Table 2) [8]. This arrhythmogenic diathesis becomes particularly concerning during pregnancy. The gestational period naturally lowers the arrhythmic threshold due to increased sympathetic activity, hormonal fluctuations, and myocardial stretch secondary to volume expansion. Among the historical cohort of pregnant patients, the case reported by Ding et al. required the implantation of an ICD, underscoring the severity of this risk in the peripartum setting.

Although our adult patient (Case 2) safely navigated pregnancy and the puerperium without arrhythmic events, the presence of extensive apical and inferior LGE dictates mandatory, long-term rhythm surveillance. Furthermore, the persistent repolarization abnormalities (diffuse T-wave inversions) observed in our pediatric patient (Case 1) suggest that the electrical instability associated with the dysplastic apex may manifest early in life. Consequently, Holter monitoring may be considered in the management of ILVAH patients, regardless of their current symptomatic status or age.

In our comprehensive review of the 59 reported cases, 19 patients experienced documented atrial arrhythmias (predominantly atrial fibrillation and flutter) [8,13,15,17,27,31,33,36,39], while 5 patients presented with life-threatening ventricular arrhythmias or sudden cardiac arrest [6,36,37,39]. Although these figures may be influenced by publication bias, they unequivocally highlight a substantial arrhythmic burden.

### 4.5. Etiopathogenetic Hypotheses

The exact embryological etiology of ILVAH remains entirely speculative. Historically, the hallmark features of the disease have been interpreted as primary developmental anomalies. Furthermore, the occasional presence of a hyper-trabeculated endocardium has led to instances of misclassification as primary left ventricular non-compaction. However, aligning with the current understanding that LVNC is often a phenotypic consequence of altered hemodynamics rather than a distinct cardiomyopathy, we propose two non-mutually exclusive pathways.

The first hypothesis suggests a primary disorder of ventricular morphogenesis. Ventricular apical differentiation begins during early cardiac looping (between the fourth and eighth embryonic weeks). As the heart tube undergoes rightward looping (D-looping), the primitive ventricle initiates a phase of intensive trabeculation and expansion. This morphogenetic “ballooning” of the apical zone is a strictly flow-dependent process; the mechanical shear stress and pressure exerted by intracardiac hemodynamics act as critical epigenetic signals for the longitudinal elongation and conical shaping of the ventricular cavity. In this model, ILVAH arises from a localized failure of this apical remodeling. Unlike Hypoplastic Left Heart Syndrome (HLHS)—where a global failure of flow-dependent growth leads to generalized hypoplasia—ILVAH preserves valvular and mid-cavity structures, pointing to a specific disruption of geometric remodeling during late cardiac morphogenesis.

The second hypothesis, fueled by our unprecedented fetal data, suggests a secondary morphological arrest triggered by a focal insult. Our Case 1 provides a crucial temporal window into this mechanism: the presence of a distinct, echogenic triangular mass at 20 weeks’ gestation effectively isolated the LV apex and abruptly stopped intracavitary flow. We hypothesize that an early focal event—be it an involuting tumor (e.g., rhabdomyoma), a fibro-fatty hamartoma, or localized ischemic calcification—acts as a physical “plug.” By disrupting the local hemodynamics essential for apical “ballooning,” this mass mechanically prevents the physiological longitudinal growth of the LV cavity. The characteristic elongation and “wrapping” of the RV would then be a secondary, compensatory adaptation to the available pericardial space during the third trimester. Over time, as observed in our 34-week scan, the initial lesion integrates into the myocardium, maturing into the fibro-fatty apical plug observed postnatally. This “common final pathway” explains why patients with different genetic backgrounds (e.g., *LMNA* or *NEXN* mutations) eventually converge on the same distinctive phenotype.

## 5. Limitations

The findings of this comprehensive review must be interpreted in light of several inherent methodological limitations. First, our conclusions are derived entirely from case reports and small case series, which are intrinsically susceptible to publication bias. It is highly probable that severe, symptomatic presentations or acute decompensations are overrepresented in the literature, while asymptomatic individuals with ILVAH may remain undiagnosed or unreported. Consequently, the true natural history and exact prevalence of complications cannot be definitively established. Second, this level of evidence precludes the determination of strict causal relationships between the anatomical anomaly and all reported clinical events. Prospective, multicenter registries are required to accurately define the clinical spectrum and optimal management of ILVAH.

## 6. Conclusions

Although often described as a benign condition, Isolated Left Ventricular Apical Hypoplasia may not invariably follow a completely asymptomatic course. Our cases, together with the available literature, suggest that the remodeled LV apex could represent a potential substrate for mechanical and electrical vulnerability.

Consequently, the management of ILVAH mandates a proactive, life-long approach. This should include serial echocardiographic monitoring, multidisciplinary peripartum planning, and rhythm surveillance to mitigate the intrinsic arrhythmogenic risk posed by apical fibrosis.

## Figures and Tables

**Table 1:** Comprehensive overview of the analyzed ILVAH cohort (n = 59), detailing baseline demographics, differential diagnoses, associated structural anomalies, heart failure status, therapeutic interventions, and clinical outcomes.

Case (n)	Study	Sex	Age (Years)	Differential Diagnosis	Structural Anomalies	Symptomatic HF	LVEF	HF Treatment	Antiarrhythmic Treatment	Outcome
1–3	Fernandez-Valls et al. (2004) [1]	F (2/3)	22/26/46	Idiopathic cardiomyopathy		Yes (3/3)	30/40/45	standard		Survived (3/3)
4	Marin et al. (2007) [7]	M	0.25	RV aneurysm		No		N/A		Survived
5	Flett et al. (2008) [4]	F	37	postpartum cardiomyopathy	mitral prolapse	Yes		standard	amiodarone, coumadin	Survived
6	Irving et al. (2009) [8]	M	19	N/A		Yes	20	standard		Deceased
7	Meléndez et al. (2010) [9]	F	9	RV diverticula		No		N/A		N/A
8–9	Patrianakos et al. (2010) [5]	F (2/2)	11/35	postpartum cardiomyopathy	mitral prolapse	Yes (1/2)		standard		Survived (2/2)
10	Motwani et al. (2011) [10]	M	63			Yes			Elective cardioversion	Survived
11	Haffajee et al. (2011) [11]	M	50		PDA	Yes	30	standard		Survived
12	Vanhecke et al. (2011) [12]	F	53	Idiopathic cardiomyopathy		Yes	45	standard		Survived
13	Chaowu et al. (2011) [13]	F	22		Complete transposition of the AV valves, PDA	Yes		N/A		Survived
14–20	Yan et al. (2012) [14]	F (4/7)	6–44			(2/7)	47 ± 17	N/A		N/A
21	Tumabiene et al. (2012) [15]	F	21	Idiopathic cardiomyopathy		Yes	52	standard		Survived
22	Ong et al. (2012) [16]	F	11			No	50			Survived
23	Starmer et al. (2012) [17]	M	62			Yes	20	standard		Survived
24	Moon et al. (2013) [18]	M	33		pulmonary and aortic stenosis	No	60			Survived
25	Pica et al. (2014) [19]	M	22			Yes		standard		N/A
26	Orsborne et al. (2014) [20]	F	17			Yes		N/A		N/A
27	Braga et al. (2014) [21]	F	66	ARVC		Yes	48	N/A		Survived
28–29	Baroni et al. (2014) [22]	F (1/2)	12/45	congenital amyoplasia/LV non-compaction	ASD I (1/2)	Yes (1/2)	30/50	standard		Survived
30	Zhao et al. (2015) [23]	M	19			No	52	standard		Survived
31	Mirdamadi et al. (2016) [24]	N/A	19	N/A		Yes	60			Survived
32	Alizadeh Sani et al. (2016) [25]	M	13			Yes	35	standard		Survived
33	Ding et al. (2016) [6]	F	22	Postpartum cardiomyopathy		Yes	10	standard	coumadin, ICD	Survived
34	Hong et al. (2016) [26]	F	34	Dilated cardiomyopathy		Yes	44	standard		Survived
35–39	Meng et al. (2013) [27]	F (2/5)	5 (3–24)		PDA (2/5)	Yes (2/5)	34/60/60/60/60	N/A		Survived (5/5)
40	Skidan et al. (2019) [28]	M	32	Idiopathic heart failure	myocardial non-compaction	Yes	32	standard	digitalis, amiodarone, rivaroxaban, ICD, ablation	Survived
41	Schapiro et al. (2019) [29]	M	17	HLHS/ARVC		Yes				Survived
42	Choh et al. (2020) [30]	M	2			Yes	60	standard	carvedilol	Survived
43	Dattani et al. (2021) [31]	F	64	mural thrombus		Yes	45	standard	amiodarone, CRT-P, AV nodal ablation	Survived
44	Ramamurthy et al. (2021) [32]	M	1.25	Dilated cardiomyopathy		No	55			Survived
45–46	Liao et al. (2021) [33]	F (1/2)	2/18	Dilated cardiomyopathy		Yes (1/2)	27/71	standard		Survived (2/2)
47	Marinelli et al. (2021) [34]	F	52	ARVC		No	60			Survived
48	Maidman et al. (2023) [35]	M	58	abnormally rotated heart		Yes	45	N/A		Survived
49	Manso et al. (2021) [36]	M	13	N/A		No	51			Survived
50	Román et al. (2023) [37]	M	24	tachycardia-induced cardiomyopathy		Yes	17	standard	carvedilol, digoxin, coumadin	Survived
51	Şahin et al. (2023) [38]	F	22	LV non-compaction		No	60	N/A		Survived
52–53	Andrade-Cuellar et al. (2024) [39]	F (1/2)	10/23		Aneurysmal ASD	Yes	42	standard + PH treatment	cryoablation/ICD	Survived
54	Liao et al. (2024) [2]	M	73	Idiopathic heart failure	mitral/tricuspidal dysplasia	Yes	60	standard + surgery		Survived
55	Hu et al. (2025) [40]	M	32	dilated cardiomyopathy	IVA hypoplasia	Yes	41	standard		Survived
56	Kolaparthi et al. (2025) [41]	M	40	dilated cardiomyopathy/ARVC		No	53	standard		Survived
57	Ojha et al. (2025) [42]	M	5	idiopathic cardiomyopathy		Yes		N/A		Survived
**58**	**Case 1**	**M**	**0**	**VSD, idiopathic cardiomyopathy**	**VSDs**	**Yes**	**45**	**standard**		**Survived**
**59**	**Case 2**	**F**	**17**	**Idiopathic** **cardiomyopathy**		**Yes**	**55**	**standard**		**Survived**

**Table 2:** Spectrum of baseline electrocardiographic (ECG) abnormalities and incidence of reported arrhythmic events (tachyarrhythmias and bradyarrhythmias) in the analyzed ILVAH cohort (n = 59). ECG = electrocardiography. Denominators vary across parameters due to the incomplete reporting of specific electrocardiographic features or the lack of published ECG traces in the original historical case reports.

Variable	Patients (n, %)
Baseline ECG	48/59 (81.4)
Normal ECG	6/48 (12.5)
Sinus Rhythm	34/48 (70.8)
Q Wave	4/48 (8.3)
Poor R-wave progression	15/48 (31.2)
QRS anomalies (low voltage. fractionated QRS. left branch bundle block)	16/46 (34.8)
T-wave anomalies	14/47 (29.8)
*Arrhythmias*	*33/52 (63.5)*
*Bradyarrhythmias*	
Sinus Bradycardia	2/52 (3.8)
*Tachyarrhythmias*	
Atrial Fibrillation	17/52 (32.7)
Atrial Flutter	2/52 (3.8)
Ventricular Tachycardia	1/52 (1.9)
Ventricular Fibrillation	1/52 (1.9)
Non-sustained ventricular tachycardia	3/52 (5.8)
*Unspecified arrhythmias*	*7/52 (13.5)*

**Table 3:** Comparison of the clinical trajectories of pregnant patients with ILVAH. The table details maternal age, timing of diagnosis, antepartum status, delivery mode, postpartum clinical presentation, echocardiographic findings, and therapeutic management.

Cases (n)	Study	Age (Years)	Antepartum Diagnosis	Differential Diagnosis	Antepartum Symptoms	Delivery	Postpartum Symptoms	Postpartum Cardiac US	Minimum LVEF (%)	Treatment
1	Flett et al. (2008) [4]	37	No	peripartum cardiomyopathy	No	N/A	pulmonary edema	ILVAH	N/A	Standard HF treatment, amiodarone, warfarin
2	Ding et al. (2016) [6]	22	No	pulmonary emboli/peripartum cardiomyopathy	N/A	N/A	lethargy, dyspnea	apical thrombus	10	Standard HF treatment, warfarin, ICD
3	Patrianakos et al. (2010) [5]	35	No	peripartum cardiomyopathy	N/A	N/A	pulmonary edema	**restrictive filling pattern, atrial dilatation**	40	Standard HF treatment, amiodarone, warfarin
**4**	**Case 2**	**17**	**Yes**	**dilated cardiomyopathy**	**No**	**cesarean section**	**Mild**		**50**	**furosemide, low-dose enalapril**

## Data Availability

The original contributions presented in this study are included in the article. Further inquiries can be directed to the corresponding authors.

## Abbreviations

The following abbreviations are used in this manuscript:

ILVAH	Isolated Left Ventricular Apical Hypoplasia
CMR	Cardiac Magnetic Resonance
LV	Left Ventricle
HF	Heart Failure
VSD	Ventricular Septal Defect
LVEF	Left Ventricular Ejection Fraction
RV	Right Ventricle
NTproBNP	N-terminal pro B-type natriuretic peptide
LAVi	Left Atrial Volume Index
GLS	Global Longitudinal Strain
TAPSE	Tricuspid Annular Plane Systolic Excursion
ECG	Electrocardiogram
LGE	Late Gadolinium Enhancement
NYHA	New York Heart Association
PH	Pulmonary Hypertension
LA	Left Atrium
ICD	Implantable Cardioverter Defibrillator
PAP	Pulmonary Arterial Pressure
